# Generation of *Mycoplasma hominis* gene-targeted mutants by targeting-induced local lesions in genomes (TILLING)

**DOI:** 10.1186/s12864-018-4917-1

**Published:** 2018-07-09

**Authors:** S. Pereyre, C. Bénard, C. Brès, C. Le Roy, J. P. Mauxion, F. Rideau, P. Sirand-Pugnet, B. Henrich, C. Bébéar

**Affiliations:** 1 USC-EA 3671 Mycoplasmal and Chlamydial Infections in Humans, University of Bordeaux, Campus Bordeaux Carreire, 146 rue Léo Saignat, 33076 Bordeaux Cedex, France; 2 USC-EA 3671 Mycoplasmal and Chlamydial Infections in Humans, INRA, Bordeaux, France; 3 Bacteriology department, French National Reference Center for bacterial STI, CHU Bordeaux, Bordeaux, France; 4 UMR 1332 BFP, INRA, Univ. Bordeaux, F-33140 Villenave d’Ornon, France; 5 Bordeaux Genome-Transcriptome Facility, F-33610 Cestas, France; 6 Institute of Medical Microbiology and Hospital Hygiene, Medical Faculty of the Heinrich-Heine-University Duesseldorf, Düsseldorf, Germany

**Keywords:** *Mycoplasma hominis*, Chemical mutagenesis, Ethyl methanesulfonate, TILLING

## Abstract

**Background:**

*Mycoplasma hominis* is a human urogenital pathogen involved in gynaecological, neonatal and extra-genital infections. However, no versatile genetic tools are currently available to study the pathogenicity of this bacterium. Targeting-Induced Local Lesions IN Genomes (TILLING) is a reverse-genetic method that combines point mutations induced by chemical mutagenesis with a DNA screening technique. We used ethyl methanesulfonate (EMS) that introduces C-G to T-A transition mutations to generate a library of *M. hominis* mutants. As a proof of concept, mutagenized organisms were screened for mutations in two target genes previously associated with the mycoplasma pathogenicity, the *vaa* gene encoding an adhesin lipoprotein and the *oppA* gene encoding the main *ecto*ATPase of the bacterium. The resulting mutants were evaluated using functional assays, an adhesion to HeLa cell assay for *vaa*-mutants and an ATPase activity test for *oppA*-mutants.

**Results:**

A 1200-clone library was generated by exposing *M. hominis* PG21 to 9 mg/mL EMS for 3 h. To identify mutants of interest, targeted gene fragments were amplified, heat-denatured, slowly reannealed and digested with the mismatch-specific endonuclease ENDO1. If multiple alleles were present in the PCR amplicons, these alleles formed heteroduplexes during reannealing that were specifically cleaved by ENDO1 at mismatching positions.

A total of four *vaa*-mutants and two *oppA*-mutants harbouring missense mutations were obtained and fully sequenced. Zero to eight additional mutations were identified in the genomes of each mutant. The *vaa*-mutants were tested for adhesion to immobilized HeLa cells but their adhesion was not significantly different from the adhesion of *M. hominis* PG21. One of the two *oppA*-mutants that were tested for ATPase activity presented a higher affinity for its ATP substrate than the parental strain.

**Conclusion:**

For the first time, we demonstrated that *M. hominis* gene-targeted mutants could be successfully obtained using this TILLING strategy. In the absence of robust genetic tools for studying *M. hominis*, the TILLING strategy that can target any gene of the genome could help to elucidate gene functions and to better understand the pathogenesis of this human pathogenic species.

## Background

*Mycoplasma hominis* is a human urogenital pathogen involved in gynaecological, neonatal and extra-genital infections [1]. The genome of the reference strain, *M. hominis* PG21, is the second smallest genome among self-replicating free-living organisms [2]. Although a conjugal transfer of the transposon Tn*916* from *Streptococcus faecalis* to *M. hominis* was reported 30 years ago [3], no versatile genetic tools are currently available for this species. Whereas *Mycoplasma arthritidis,* a phylogenetically closely related species belonging to the same Hominis group, has successfully been transformed using the PEG method [4], all attempts to transform *M. hominis* using either PEG or electroporation methods, as well as transposons, suicide plasmids or replicative plasmids have failed. Only one study reported the transformation of *M. hominis* by the plasmid pAM120 using electroporation in the 2000s [5], but the experiments could not be reproduced. A cutting-edge synthetic biology approach, which consists of cloning and subsequent engineering of *M. hominis* genome in yeast is currently under development but the back transplantation of the genome from yeast to a recipient bacterium has not been achieved yet [6]. The lack of genetic engineering tools for *M. hominis* has limited our capacity to modify its genome in order to elucidate gene functions and to understand its pathogenesis.

Chemical mutagenesis methods may offer an interesting alternative method to modify the genome of this intractable species. Targeting-Induced Local Lesions IN Genomes (TILLING) combines point mutations induced by standard chemical mutagenesis with a sensitive DNA screening technique that identifies single nucleotide polymorphisms (SNPs) in the targeted gene. This reverse-genetic strategy has mainly been used in plants such as *Arabidopsis thaliana* [7], tomato [8] and *Cucurbita pepo* [9] but also in parasites such as *Toxoplasma gondii* [10]. Only one publication reported its use in a bacterium, the intracellular *Chlamydia trachomatis* species [11].

In this study, we used ethyl methanesulfonate (EMS) that introduces C-G to T-A transition mutations to generate a library of *M. hominis* mutants. As a proof of principle, mutagenized organisms were screened for mutations in two target genes, the *vaa* gene encoding the variable adherence-associated (Vaa) adhesin lipoprotein [12] and the *oppA* gene encoding the substrate binding subunit of an oligopeptide permease and the main *ecto*ATPase of the bacteria [13, 14]. The generated *vaa*- and *oppA*-mutants were fully sequenced and evaluated using two functional assays, an adhesion test to HeLa cells for *vaa*-mutants and an ATPase activity test for *oppA*-mutants.

## Results

### Generation of a *M. hominis* mutant library

**Fig. 1:**
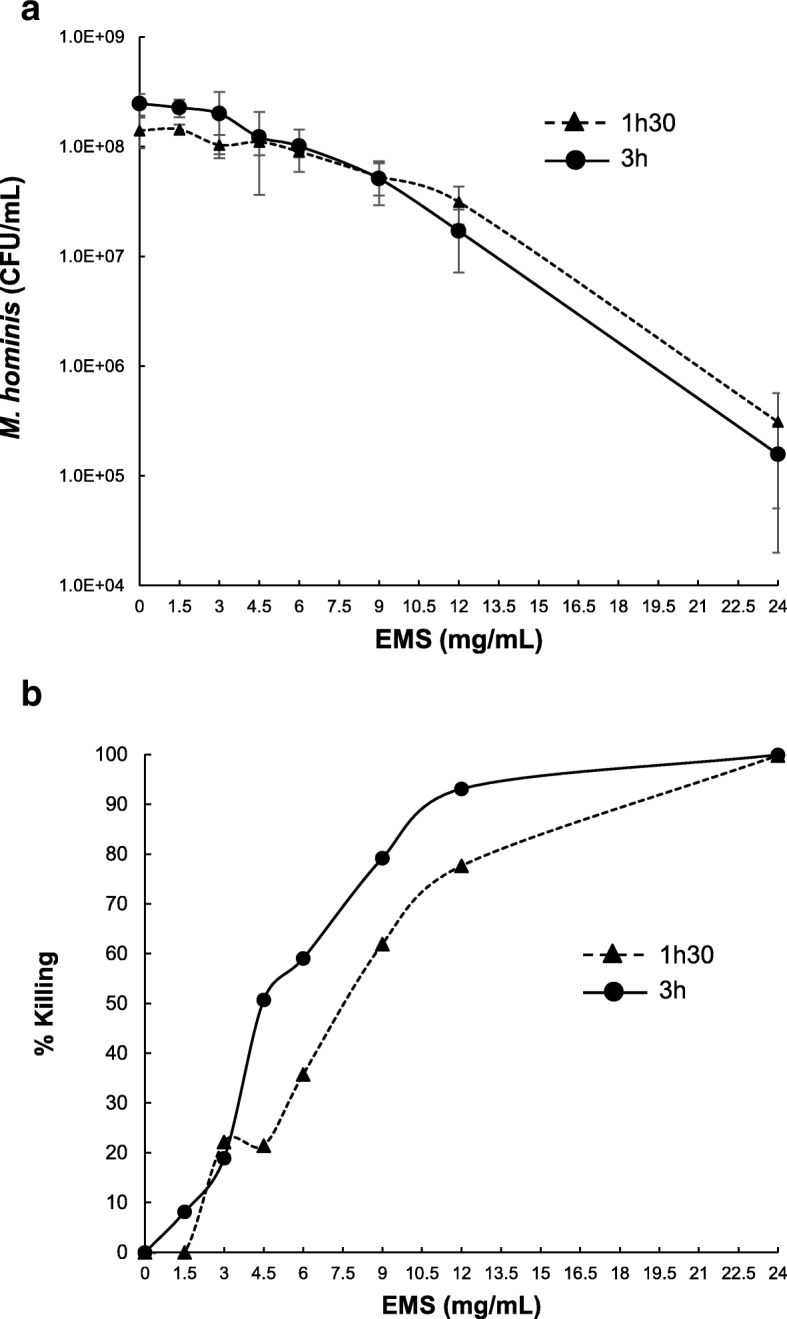
Effects of EMS mutagenesis on *M. hominis* survival. Concentrations of *M. hominis* PG21 were determined after treatment with various concentration of EMS for 1.5 h and 3 h (**a**). Three independent experiments were performed. The percentage of killing was calculated (**b**). In accordance with the literature, a 75% killing rate corresponding to exposure to approximately 9 mg/ml EMS for 3 h was chosen to generate the *M. hominis* PG21 mutant library

The effect of EMS on *M. hominis* survival was assessed after contact times of 1.5 h and 3 h (Fig. 1). A positive correlation was observed between the concentration of EMS and the killing of the bacteria. The contact time with EMS also slightly affected the survival of *M. hominis*. For the generation of the mutant library, a concentration of 9 mg/ml EMS applied for 3 h that resulted in 75% killing was chosen according to the bibliography [11]. After culture filtration to avoid bacterial aggregates and plating on agar medium, 1200 individual colonies were picked, grown and frozen.

### Screening of the *M. hominis* mutant library for *vaa*- and *oppA*-mutants

**Fig. 2:**
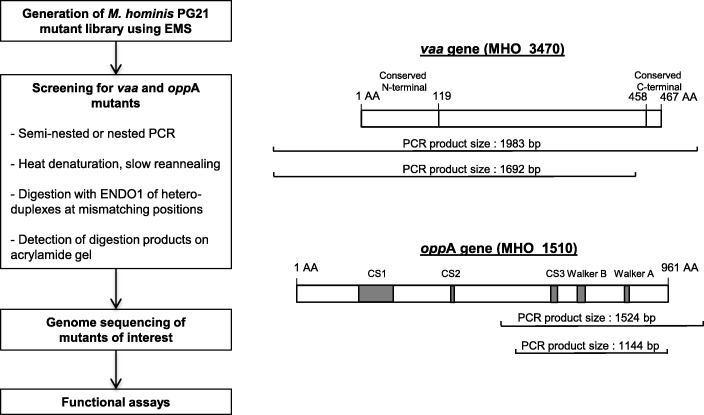
Schematic representation of the TILLING strategy used in this study

To screen the library for *vaa*-mutants, a 1692-bp PCR fragment of the *M. hominis* genome encompassing the 1295 nucleotides at the 5′-end of the *vaa* gene (MHO_3470, 1416 bp [2]) was targeted (Table 1, Fig. 2). To screen for *opp*A-mutants, a 1144-bp PCR fragment of the *oppA* gene encompassing the regions encoding the CS3, Walker B and Walker A domains involved in ATPase activity [14] of the OppA protein (MHO_1510, 2886 bp) was amplified (Table 1, Fig. 2).

**Table 1:** Oligonucleotides used in this study

Primer target and designation	Primer sequence	Product size (bp)
*vaa* gene^a^		
VaaPG21-FE	5’-AATGGGATAGTTAGTAAAGTCGG-3’	1983
VaaPG21-RE	5’-AACCCTACCATTGTCGCTAAGA-3’	
VaaPG21-FE-IRD700	5’-IRD700-AATGGGATAGTTAGTAAAGTCGG-3’	1692
VaaPG21-RI-IRD800	5’-IRD800-GAAAAATCTCCTCGTGCTGATG-3’	
*oppA* gene		
OppAPG21-FE	5’-GACAGTGGAAAAGCAGACAC-3’	1524
OppAPG21-RE	5’-AGGAACTAAAATGTCCGGGT-3’	
OppAPG21-FI-IRD700	5’-IRDye700- CATCTTCTGGCCAAGCAACT − 3’	1144
OppAPG21-RI-IRD800	5’-IRDye800-CAGTCTTGGTATGAATCAAC-3’	

^a^VaaPG21-FE and VaaPG21-RE primers are located upstream and downstream the *vaa* gene, respectively

After the screening procedure using ENDO1 nuclease, a total of six *vaa* and four *oppA M. hominis* mutants were obtained (Table 2). Four mutants were discarded: (i) two *vaa*-mutants that harboured a C to T transition located upstream of the *vaa* gene (these mutants were initially screened because the amplified DNA fragment encompassed 397 nucleotides upstream of the gene) and (ii) two *oppA*-mutants that harboured a G181068A and a G180439A silent substitution, respectively. Overall, four *vaa*-mutants (Mho66, Mho119, Mho787 and Mho1133) and two *oppA*-mutants (Mho369 and Mho940), each harbouring one amino-acid substitution in the targeted region of the *vaa* and *oppA* genes, respectively, were selected. The genomes of these six selected mutants were fully sequenced. The expected mutation was found in every mutant and zero to eight additional mutations were retrieved in the whole genome of each mutant (Table 3). Notably, mutant Mho66 harboured no additional mutation and mutant Mho369 harboured only one additional mutation in an intergenic region.

**Table 2:** *vaa*- and *oppA*-mutants obtained by screening the 1200 EMS-generated *M. hominis* clone library

Mutant designation	Targeted gene	Nucleotide substitution^a^	Amino-acid substitution^b^	Comments
Mho66	*vaa*	G410726A	Glu54Lys	
Mho119	*vaa*	C411332T	Leu328Phe	
Mho787	*vaa*	C410526A	Pro59His	
Mho864	*vaa*	C410197T	none	Mutation located upstream of the *vaa* gene (intergenic region)
Mho1055	*vaa*	C410154T	none	Mutation located upstream of the *vaa* gene (intergenic region)
Mho1133	*vaa*	G410726A	Gly126Ser	
Mho258	*oppA*	G181068A	Leu813Leu	Silent mutation
Mho369	*oppA*	G180439A	Asp604Asn	
Mho785	*oppA*	C181239T	Asp870Asp	Silent mutation
Mho940	*oppA*	G180634A	Glu669Lys	

^a^Nucleotide substitutions are numbered according to the *M. hominis* PG21 genome (GenBank accession number FP236530)

^b^Amino acid substitutions are numbered from the N-terminal end of the targeted protein

**Table 3:** Mutations harboured by EMS-generated *M. hominis* mutants in comparison to the sequence of the *M. hominis* PG21 strain not exposed to EMS

Mutant designation	Expected mutation^a^ (gene, position in the gene)	Other mutations in the genome^a^ (Locus, gene)	Number of additional mutations
Mho66	G410726A (*vaa*, 376)	none	0
Mho119	C411332T (*vaa,* 982)	- C230929T (intergenic) - C316577T (MHO_2640, CHP) - G463593A (MHO_3770, *licA*, Protein LicA homolog) - G494526A (MHO_3990, *nusG*, Transcription antitermination protein)	4
Mho787	C410526A (*vaa,* 176)	- C5000T (MHO_0050, *dnaN*, DNA polymerase III beta chain) - C44025T (MHO_0340, *ktrA*, Potassium uptake protein KtrA) - C172081T (MHO_1460, *rpsG*, 30S ribosomal protein S7) - C258548T (MHO_2150, *rpoD*, RNA polymerase sigma factor) - C283090T (MHO_2320, ABC transporter, ATP-binding protein) - G288972A (MHO_2370, tRNA/rRNA methyltransferase) - C441828T (MHO_3650, *asnA,* Aspartate-ammonia ligase) - C571686T (MHO_4600, *pyrH*, Uridylate kinase)	8
Mho1133	G410726A (*vaa*, 376)	- G179389A (MHO_1510, *oppA*) - C206418T (MHO_1700, *recU*, Recombination protein U homolog) - C381191T (MHO_3200, CHP, Hypothetical lipoprotein) - G462316A (MHO_3760, *gyrB*, DNA gyrase subunit B) - G490546A (intergenic) - C644067T (MHO_5190, *pip*, Proline iminopeptidase)	6
Mho369	G180439A (*oppA,* 1813)	- G230779A (intergenic)	1
Mho940	G180634A (*oppA,* 2008)	- G136127A (MHO_1080, ATB-binding protein) - G138607A (MHO_1090, HP) - G203092A (MHO_1670, *cmk*, Cytidylate kinase) - G211597A (MHO_1740, *oppB*, Oligopeptide transport system permease protein) - A227310G (MHO_1880, *aspS*, Aspartyl-tRNA synthetase) - G357674A (MHO_3060, *leuS*, Leucyl-tRNA synthetase) - G371725A (MHO_3130, *atpA*, F1-likeX0) - G649646C (MHO_5220, *hsdS*, Type I restriction enzyme specificity protein)	8

^a^mutations are numbered according to the *M. hominis* PG21 genome (GenBank accession number FP236530)

*CHP* conserved hypothetical protein, *HP* hypothetical protein

### Functional assays applied to *vaa*- and *oppA*-mutants

**Fig. 3:**
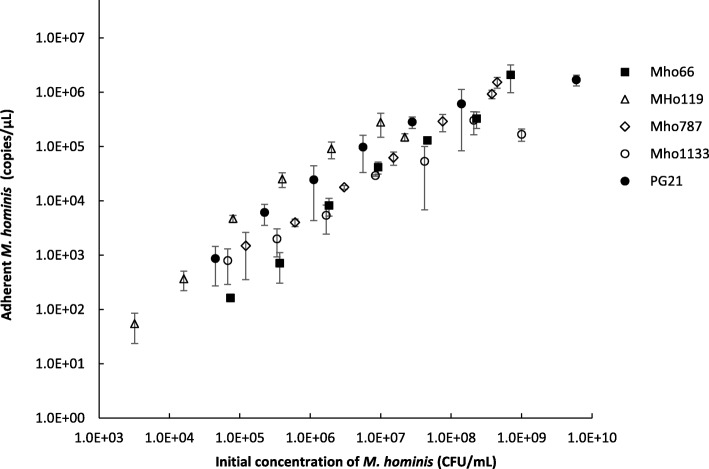
Adhesion to HeLa cells of EMS-generated *M. hominis vaa*-mutants. *M. hominis* cells were added to immobilized HeLa cells in triplicate and incubated 4 h at 37 °C. After removal of unbound *M. hominis* cells by washing, the adherent *M. hominis* cells were quantified by real-time PCR. One representative experiment of three is shown

The four *vaa*-mutants, Mho66, Mho119, Mho787 and Mho1133, and the parental PG21 *M. hominis* strain, which has a functional in-frame *vaa* gene, were tested for adhesion to HeLa cells immobilized on microtiter plates to quantify bacterial adherence. In all cases, the number of adherent *M. hominis* cells increased with the amount of *M. hominis* inoculum used in the assay (Fig. 3). The adhesion of the four *vaa*-mutants and the non-mutated *M. hominis* PG21 strain was not significantly different (*p* = 0.73, Kruskal-Wallis test).

**Fig. 4:**
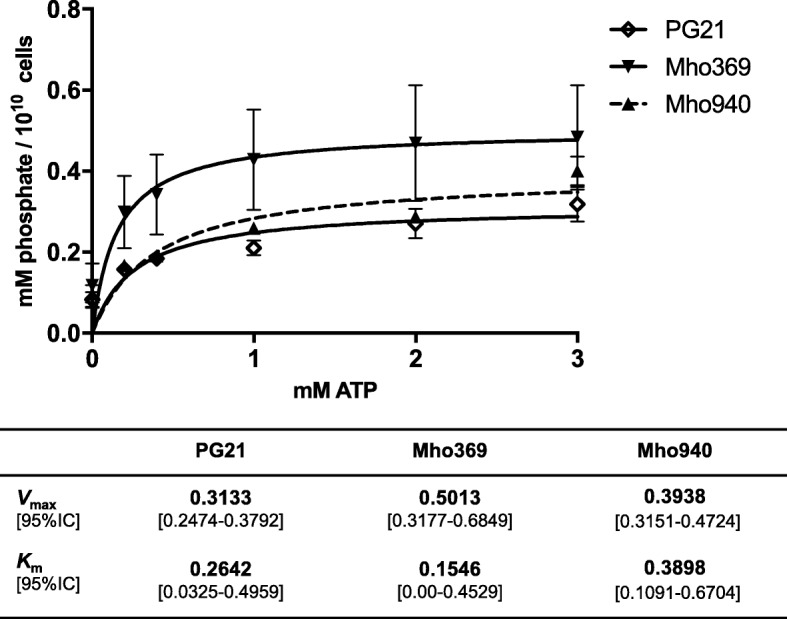
*ecto*ATPase activity of EMS-generated *M. hominis oppA*-mutants. *ecto*ATPase activity of EMS-generated Mho369 and Mho940 *M. hominis* mutants and of *M. hominis* PG21 parental strain was determined by measuring the release of free phosphate per 10^10^ cells as a function of the ATP concentration. The data represent the means and standard errors of five (Mho369) and four (Mho940 and PG21) independent experiments with triplicate samples in each experiment. *V*_max_, maximum rate, *K*_m_, Michaelis-Menten constant, 95% IC, 95% confidence interval

The two *oppA-*mutants, Mho369 and Mho940, and the parental PG21 strain were tested for ATPase activity (Fig. 4) during which the kinetics of ATP hydrolysis were analysed by measuring the release of free phosphate [14]. Both mutants had a higher *V*_max_ than *M. hominis* PG21 and the Mho369 mutant had the lowest Michaelis-Menten *K*_m_ constant, although the 95% confidence intervals were overlapping.

## Discussion

Because the transformation of *M. hominis* PG21 has so far been elusive, chemical mutagenesis using EMS appears to be an alternative option to create gene-targeted mutants. In several organisms, a positive correlation was observed between the concentrations of EMS applied, the killing of the organism and the mutation frequency obtained [8, 11]. Only one study has reported the generation of EMS mutants and TILLING strategy in bacteria. Indeed, to generate *C. trachomatis* mutants using EMS, the concentration of EMS that generated a single C-G base pair mutation per genome corresponded to a 75% killing. Because we also observed a positive correlation between the concentration of EMS and the killing of *M. hominis*, we decided to choose, for the generation of the library, the concentration of EMS that killed approximately 75% of the *M. hominis* population, i.e. 9 mg/ml applied for 3 h.

Using these conditions, we demonstrated, after screening the 1200-clone library, that four and two *M. hominis* mutants were successfully generated in two targeted genes previously associated with the bacterium’s pathogenicity, the *vaa* gene involved in cytadherence [12] and the *oppA* gene involved in oligopeptide binding and the *ecto*ATPase activity [13, 15], respectively. Because the ATPase activity of the OppA protein is due mainly to the CS3, Walker B and Walker A gene regions [14], a fragment of 1144 bp encompassing these regions was chosen. Regarding the *vaa* gene, a 1692-bp fragment encompassing the 5′-end conserved region of the gene was targeted for this proof of concept experiment because an early premature stop in protein synthesis would have had the greatest effect on loss of Vaa-mediated cytoadhesion. To our knowledge, the substitutions that were found in the mutated Vaa and OppA have never been reported in the alleles present in other *M. hominis* strains.

The four *vaa*-mutants were tested for adhesion to immobilized HeLa cells [14]. Unfortunately, the adhesion of the four mutants did not show any significant difference with the adhesion of the parental *M. hominis* PG21 strain. However, because *M. hominis* harbours several adhesin proteins such as the P120 lipoprotein and the Lmp-related proteins [16], this adhesion assay may not be sensitive enough to detect a potential adhesion decrease caused by the mutation of the Vaa protein. A control strain with an inactivated Vaa protein or a phase variable “OFF” variant would be necessary to determine whether the sensitivity of this assay was accurate. Analysis of the *vaa* gene sequence of the *M. hominis* PG21 genome showed that C-G to T-A EMS-generated transitions could theoretically create 23 stop codons, leading to truncated proteins. Thus, enlarging our EMS library may generate null mutants.

Regarding the two *oppA*-mutants, mutant Mho369 was of interest because (i) it harboured only one additional mutation in an intergenic region of its genome, suggesting that the observed phenotype could be attributed to the EMS-generated *oppA* mutation with a high degree of certainty and (ii) it had a higher *V*_max_ and a lower *K*_m_ than *M. hominis* PG21, although 95% confidence intervals were overlapping. This finding suggests that Mho369 may have the capacity to hydrolyse ATP with a higher turnover. This mutant harboured an aspartic acid-to-asparagine substitution located upstream of the CS3 motif, known to be involved in the ATPase activity of the protein [14]. Further studies such as cloning, expression and analysis of the mutated OppA protein could be informative in future works. Nevertheless, although recombinant OppA-mutant proteins have previously been generated [14], *oppA*-mutated *M. hominis* cells had never been obtained before. Since OppA is involved in *M. hominis* pathogenicity [13, 15], such mutated *M. hominis* cells will allow for the characterization of the effect of single amino-acid changes in OppA on the pathogenicity of the mycoplasma.

The TILLING strategy used in this study presents some advantages and some drawbacks. The principal advantage is its capacity to generate gene-targeted mutants in species such as *M. hominis* that are difficult to genetically manipulate. The ultimate goal of the method is to generate null mutants. All genes could be potentially targeted except those genes that are essential for growth in vitro, and thus cannot be targeted for null mutations. In addition, once a library has been generated, it can be reused to search for mutants in other genes of interest. However, this method is labour-intensive, both for the generation of the library and its handling. The 1200-clone library generated in this study was not representative of the whole *M. hominis* genome and the isolation of a null mutant of Vaa and OppA was not achieved. Sequencing the whole genome of the selected mutants is necessary to check for additional mutations that could have been created in other regions of the chromosome. Indeed, the occurrence of multiple mutations in the same clone may be disruptive for functional studies. However, in a future use of the TILLING strategy, whole genome sequencing will only have to be performed on mutants presenting interesting phenotypes upon functional assays. In the present study, only one of the six sequenced mutants had no other mutation in the genome. Because one to eight additional mutations were present in the five other mutants, it is likely that the concentration and/or the EMS exposure time with *M. hominis* should be decreased slightly in future experiments. Decreasing these parameters would require screening more than 1200 clones to select for a mutant, which would increase the workload of the method. However, the technical improvement of robotic colony pickers may unlock this limitation, making the TILLING strategy a broader avenue for the functional genomic of intractable bacteria.

## Conclusion

Although genetic manipulations are still hardly achievable in *M. hominis*, targeted mutants were successfully generated using the TILLING strategy. Further experiments are needed to adjust the conditions of EMS mutagenesis to limit the mutation frequency to one mutation per genome, but this strategy will be helpful to elucidate the gene functions and to better understand the pathogenesis of this human urogenital species.

## Methods

### EMS mutagenesis of *M. hominis* PG21

The *M. hominis* PG21 reference strain (ATCC 23114) was grown in Hayflick broth supplemented with arginine (hereafter named Hayflick arginine medium) [17]. One millilitre of a culture of *M. hominis* PG21 ranging between 1 × 10^8^ and 3 × 10^8^ colony forming unit (CFU)/ml was exposed to concentrations of EMS ranging from 0 to 24 mg/ml for 1.5 h and 3 h at room temperature, including a 30-min centrifugation step at 15,000 *g*. Pellets were resuspended in 1 ml of Hayflick arginine medium, and *M. hominis* concentration was determined by plating on Hayflick agar plates to assess *M. hominis* survival.

To generate the mutant library, a 10^8^ CFU/ml *M. hominis* culture was exposed to EMS, filtered on 0.45 μm sterile mixed cellulose ester membrane (Millex-HA, Merck Millipore, Cork, Ireland), and 10^− 1^ to 10^− 4^ serial dilutions in Hayflick arginine medium were plated on Hayflick agar plates. After 48 h at 37 °C under 5% CO_2_, individual colonies were picked and grown in 1 ml of Hayflick arginine medium until the colour changed. A total of 800 μl of the latter culture was frozen at − 80 °C, and 200 μl was used for DNA extraction.

### Screening for *vaa*- and *oppA*-mutants in *M. hominis*

Two hundred microliters from four distinct clone cultures were pooled together before DNA extraction using the MagNA Pure 96 DNA and Viral NA Small Volume kit on the MagNA Pure 96 instrument (Roche Diagnostics, Meylan, France). DNA extraction was also performed on 200 μl of each of four single clone cultures when a mutated pool of clones was identified.

PCR fragments of 1692 bp and 1144 bp in regions of interest of the *vaa* and the *oppA* genes were amplified by semi-nested and nested PCR, respectively, using primers presented in Table 1. The first round of PCR was performed on a CFX96 real-time PCR detection system (Bio-Rad Laboratories, Hercules, CA, USA) in a final volume of 25 μl using 2.5 μM of tartrazine 10X buffer (50 mM Tris HCl pH 8, 500 μg/ml bovine serum albumin, 0.5% Ficoll, 1% sucrose, 30 mM KCl, 3 mM MgCl_2_, 1 mM tartrazine), 0.1 μM of each primer, 120 μM dNTP, 0.0005 U of GoTaq® qPCR Master Mix (Promega, Madison, WI, USA), and 1 μl of DNA extract. The amplification conditions were 3 min at 95 °C followed by 35 cycles of 15 s at 95 °C, 20 s at 58–68 °C, 2 min at 72 °C then 10 min at 72 °C. The second round of PCR was performed using 0.5 μl from the first PCR and IRD700- and IRD800-labelled primers (Table 1). After checking accurate amplification by agarose gel electrophoresis, 5 μl of the PCR products was used to screen for *vaa-* and *oppA*-mutants. PCR amplicons were heat-denatured 3 min at 95 °C and slowly reannealed using a temperature gradient from 95 °C to 8 °C (0.1 °C/s) before a 4 °C final step. The reannealed amplicons were digested using ENDO1 nuclease at 0.33 U/μl, prepared as previously reported [8], for 20 min at 45 °C and cooled to 4 °C to stop the enzyme activity. At this step, if multiple alleles were present in the PCR amplicons, these alleles formed heteroduplexes during reannealing that were specifically cleaved by ENDO1 at mismatching positions. The digested amplicons were purified and dried as described previously [18].

SNP screening was performed using a LiCOR® DNA analyser as previously described [19]. Pools of clones containing *vaa-* and *oppA*-mutants were identified by the appearance of lower-mass DNA digestion products after electrophoresis on a 6.5% acrylamide gel. The PCR products of *vaa* or *oppA* genes from the four individual clones of the mutated pool were sequenced by Sanger method (Eurofins Genomics, Ebersberg, Germany) to identify the mutated clone and the mutation type.

### *M. hominis* genome sequencing

The genomic DNA from four *vaa*-mutants, two *oppA*-mutants and from the *M. hominis* PG21 parental strain was extracted using NucleoBond® AXG100 columns (Macherey-Nagel, Düren, Germany) and the NucleoBond® buffer Set III (Macherey-Nagel). The genomes were sequenced using paired-end V2 2X250 bp sequencing on MiSeq Illumina apparatus (San Diego, CA, USA) after generating the genomic DNA libraries using the of Nextera XT DNA Library Preparation Kit (Illumina). About 160,000 to 200,000 paired reads were obtained for each genome. Data processing including quality check, trimming, alignment with BWA (Galaxy Version 1.2.3) and variant calling using Varscan (Galaxy Version 0.1) was performed using Galaxy at https://usegalaxy.org/ [20].

### Adhesion to HeLa cell assay

*M. hominis* adhesion to immobilized HeLa cells was performed as previously reported with some modifications [14]. A calibrated culture of each *M. hominis* mutant, with concentrations ranging between 1 × 10^8^ and 4 × 10^8^ CFU/ml, was concentrated 10 times by centrifugation and serially diluted 1:5 in Hayflick arginine medium. Fifty microliters of each dilution was incubated in triplicate with HeLa cells that had been lysine-coated on 96-well microplates in 50 μl of Dulbecco’s Modified Eagle’s medium (DMEM) supplemented with 1% fetal bovine serum (DMEM-FBS) for 4 h at 37 °C. Unbound *M. hominis* cells were removed by three washings with DMEM-FBS. Adherent *M. hominis* cells were lysed using 20 μl of proteinase K 20 mg/ml from the NucleoSpin® Tissue kit (Macherey-Nagel) for 1 h at 56 °C and DNA was extracted using the MagNA Pure 96 DNA and Viral NA Small Volume kit on the MagNA Pure 96 instrument (Roche Diagnostics). A quantitative real-time PCR targeting the *yidC* gene of *M. hominis* was then performed as previously described [21]. The non-parametric Kruskal-Wallis test was used to compare the adhesion of mutants and the PG21 parental strain. A significant *p-*value was set at < 0.05.

### ATPase activity test

The ATPase assay was conducted by incubating *M. hominis* cells with ATP and subsequently using an ammonium molybdate solution to quantify the emerging free phosphates [13]. *M. hominis* cells were collected from 10 ml of logarithmic growth culture by centrifugation (6700 *g* for 10 min at 6 °C), washed twice in buffer A (120 mM NaCl, 5 mM KCl, 20 mM Tris-HCl, pH 7.5) and resuspended in 1.2 ml of buffer A. *M. hominis* cells were quantified by Taqman PCR as published earlier [22]. Triplicates of 20 μl of mycoplasma cell suspension and 20 μl of buffer A (as negative control) were each adjusted to 0, 0.2, 0.4, 1, 2 or 3 mM ATP and immediately assayed for free phosphate (to estimate phosphate background of mycoplasma suspension) or incubated for 4 h at 37 °C.

Hydrolysis of ATP was terminated by adding 200 μl of malachite green reagent (5.72% [*w*/*v*] ammonium molybdate in 6 N HCl, 2.32% [w/v] polyvinyl alcohol, 0.0812% [w/v] malachite green, and distilled water at a ratio of 1:1:2:2). The relative absorbance of the samples was measured in relation to a blank at 620 nm (Tecan Rainbow, SLT Labinstruments, Crailsheim, Germany). Inorganic phosphate at concentrations ranging from 1 to 20 nmol was used as quantification standard. Values of ATPase activity of *M. hominis* expressed as the release of nmol phosphate were corrected for the phosphate background of ATP, adjusted to 10^10^ *M. hominis* cells and used in the Michaelis-Menten equation to calculate the values of *V*_max_ and *K*_m_ using the Graph Pad Prism 6.01 software.

## Abbreviations

*C. trachomatis*: *Chlamydia trachomatis*
CHP: Conserved hypothetical protein
EMS: Ethyl methanesulfonate
HP: Hypothetical protein
*M. hominis*: *Mycoplasma hominis*
TILLING: Targeting-Induced Local Lesions IN Genomes
